# Supplementary material for: Physics-informed deep learning characterizes morphodynamics of Asian soybean rust disease
Source: Nat Commun. 2021 Nov 5;12:6424. doi: 10.1038/s41467-021-26577-1 (PMC8571353; doi:10.1038/s41467-021-26577-1)
Supplement: Supplementary file 1 — Supplementary Information [file 41467_2021_26577_MOESM1_ESM.pdf]

Supplementary Information for

Physics-Informed Deep Learning Characterizes Morphodynamics of Asian Soybean Rust Disease

August 2021

Henry Cavanagh, Andreas Mosbach, Gabriel Scalliet, Rob Lind, Robert G. Endres*
*Corresponding author: r.endres@imperial.ac.uk

Contents

Supplementary Figures **1**
Supplementary Figure 1: Convergence of the physics-informed neural network (PINN) 1
Supplementary Figure 2: Correspondence between the landscapes and morphospace 3
Supplementary Figure 3: Correspondence between the landscapes and morphospace 4
Supplementary Figure 4: Comparison of simulation and data trajectories 6
Supplementary Figure 5: Data-driven development of the tip growth model 7
Supplementary Figure 6: Comparisons of maximum *a posteriori* probability (MAP) simulations of Model 3 with data 9
Supplementary Figure 7: Chemical structure of Compound X 10

Supplementary Notes **11**
Supplementary Note 1: *P. pachyrhizi* Imaging and Image Processing 11
Supplementary Note 2: Autoencoder and PINN Neural Networks 13
Supplementary Note 3: Three Models of Tip Growth 15
Supplementary Note 4: Possible Modes of Action 16

Supplementary Methods **16**

Supplementary References **16**

Supplementary Figures

Supplementary Figure 1: Convergence of the physics-informed neural network (PINN)

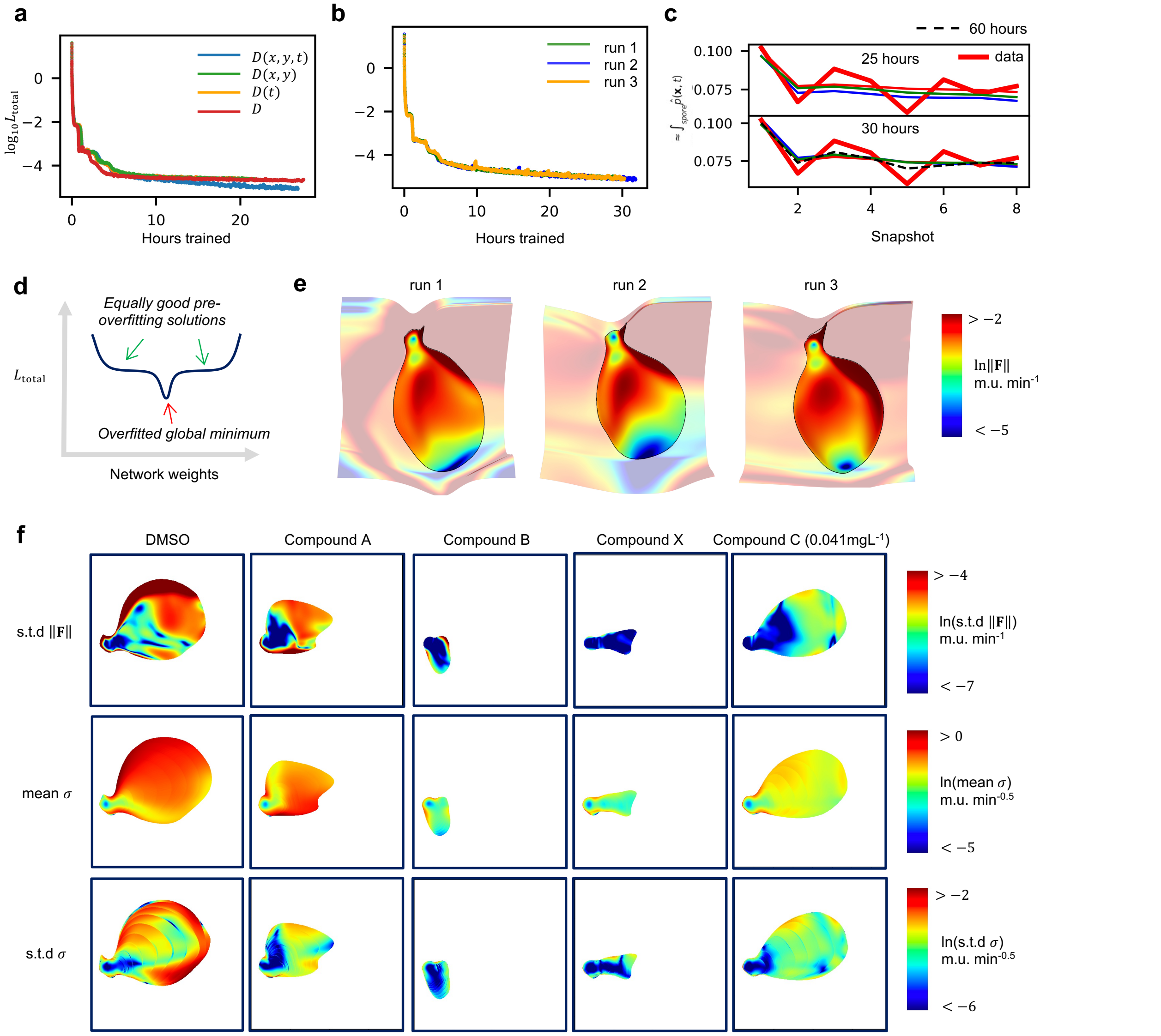

Supplementary Figure 1: **Convergence of the physics-informed neural network (PINN).** Caption continued on the following page.

Supplementary Figure 1: **Convergence of the physics-informed neural network (PINN).** (**a**) An ablation analysis comparing how L_{total} (running mean over 200 mini-batches) decreases as training progresses reveals that a diffusivity with both spatial and time dependence is the best model. The majority of the benefit likely comes from the dynamics in the spore region of morphospace, where diffusion is very high at first, and then strongly decreases such that not all spores germinate. (**b**) We repeat PINN network training three times for each condition, with different mini-batches, and L_{total} (running mean over 200 mini-batches) is shown here for each repeat of DMSO. (**c**) An approximation of the dynamics of the fraction of spores ($\approx \int_{\text{spore}} \hat{p}(\mathbf{x}, t)$) across snapshots for DMSO (with the first snapshot not shown due to its much higher fraction of spores), found by numerically integrating a box around the spore PDF peak. The data is shown in red, and the three repeats have the same coloring as in (b). The PINN first explores smooth low-frequency solutions, fitting trends common to all snapshots, before ultimately beginning to overfit to the individual snapshots, as shown for one repeat at 60 hours in black. We stop training when the PINN begins to fit to the individual snapshots, which approximately corresponds to 30, 30, 30, 20, 25 and 25 hours for DMSO and Compounds A, B, C (0.041 mgL^{-1}), C (10 mgL^{-1}) and X, respectively. (**d**) Sketch of the loss landscape, whereby the global minimum is an overfitted solution, and there may be many equally good solutions before overfitting. (**e**) The landscapes from each of the three repeats after 30 hours of training for DMSO show many common features in the central data-rich region. (**f**) For each of the conditions with significant germination (i.e. excluding Compound C at 10 mgL^{-1}), three outputs are shown: the uncertainty in the force magnitude, $\|\mathbf{F}\|$, calculated from the standard deviation across the three training repeats; the mean σ (from Eq. 1), averaged over time for the same training repeat as those of the landscapes shown in the other figures, and only calculated over regions where the PDFs are above 10^{-3}; and the uncertainty in σ, calculated in the same way as the uncertainty for $\|\mathbf{F}\|$. All outputs are expressed in terms of morphospace units, m.u. Source data are provided for (**c**).

Supplementary Figure 2: Correspondence between the landscapes and morphospace

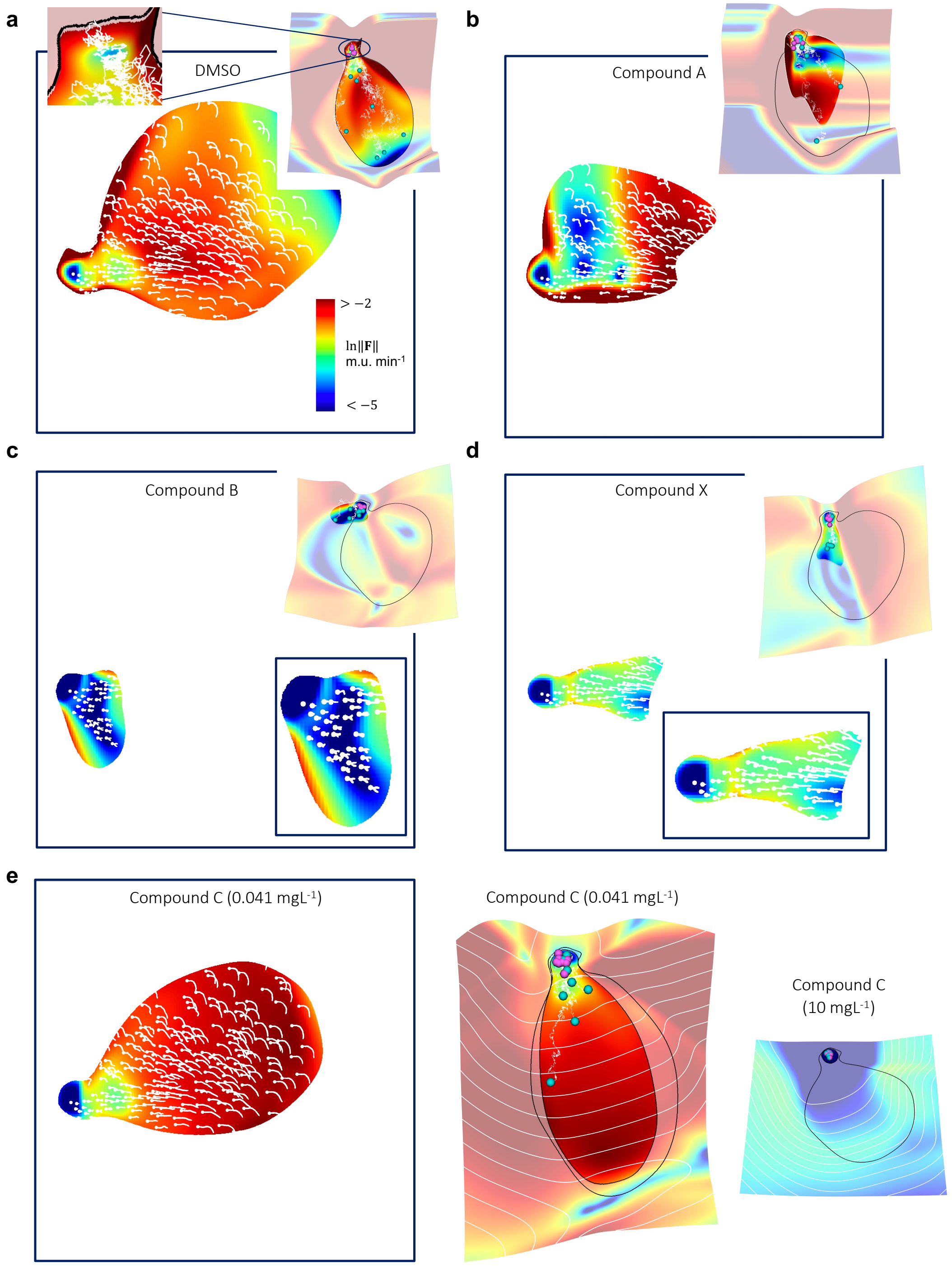

Supplementary Figure 2: **Correspondence between the landscapes and morphospace.** (**a-d**) Landscapes for DMSO and Compounds A, B and X, with samples of fungus images from the underlying morphospace, both colored by the gradient magnitude, $\|\mathbf{F}\|$, at regions where the PDFs are above 10^{-3}. (**e**) The same as described above, but for Compound C at 0.041 mgL^{-1}, alongside the landscapes for Compound C at 0.041 mgL^{-1} (with contours along equal landscape values, spaced 0.14 m.u.^{2} min^{-1} apart, where m.u. stands for morphospace units) and 10 mgL^{-1} (with contours spaced 0.02 m.u.^{2} min^{-1} apart).

Supplementary Figure 3: Correspondence between the landscapes and morphospace

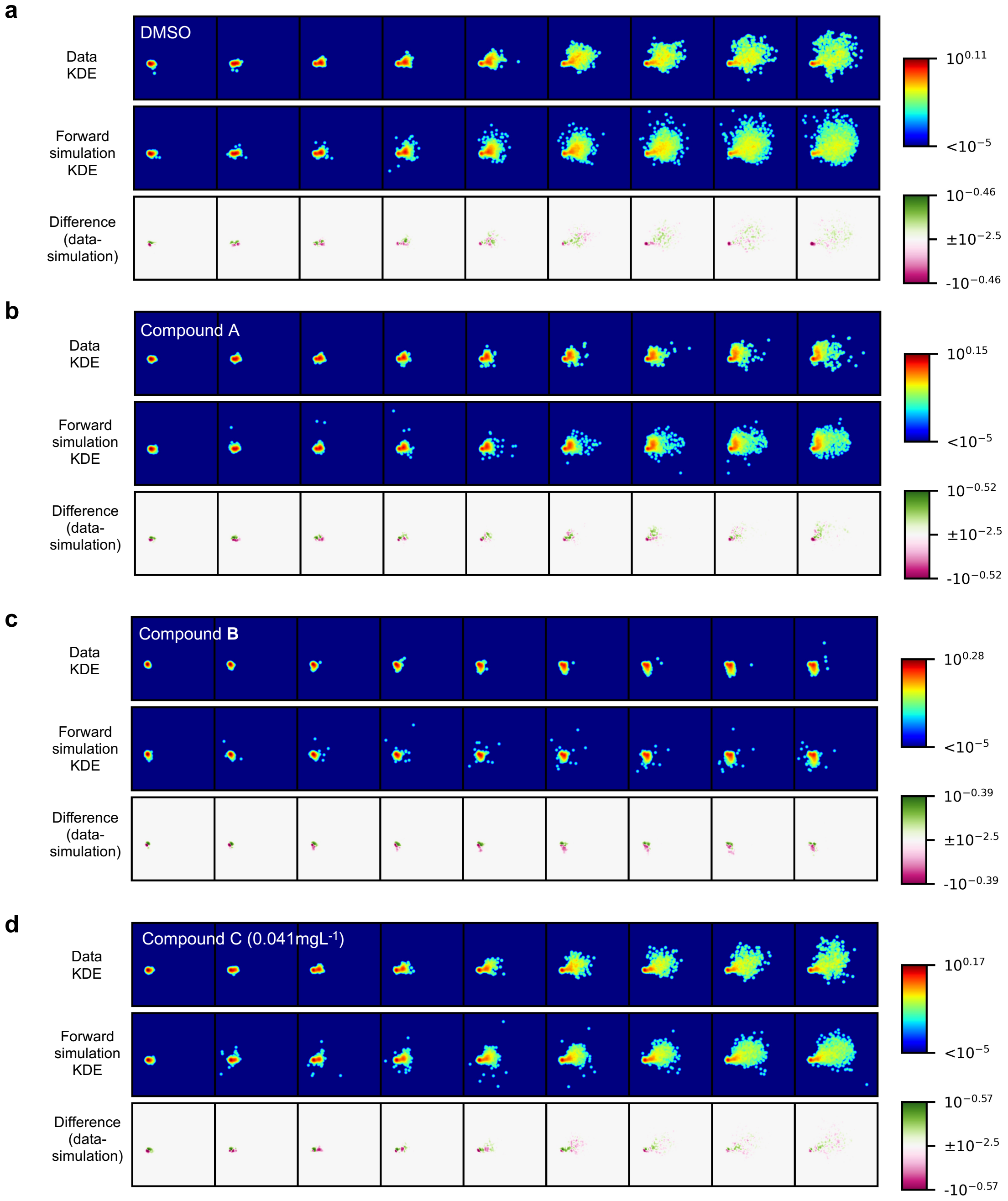

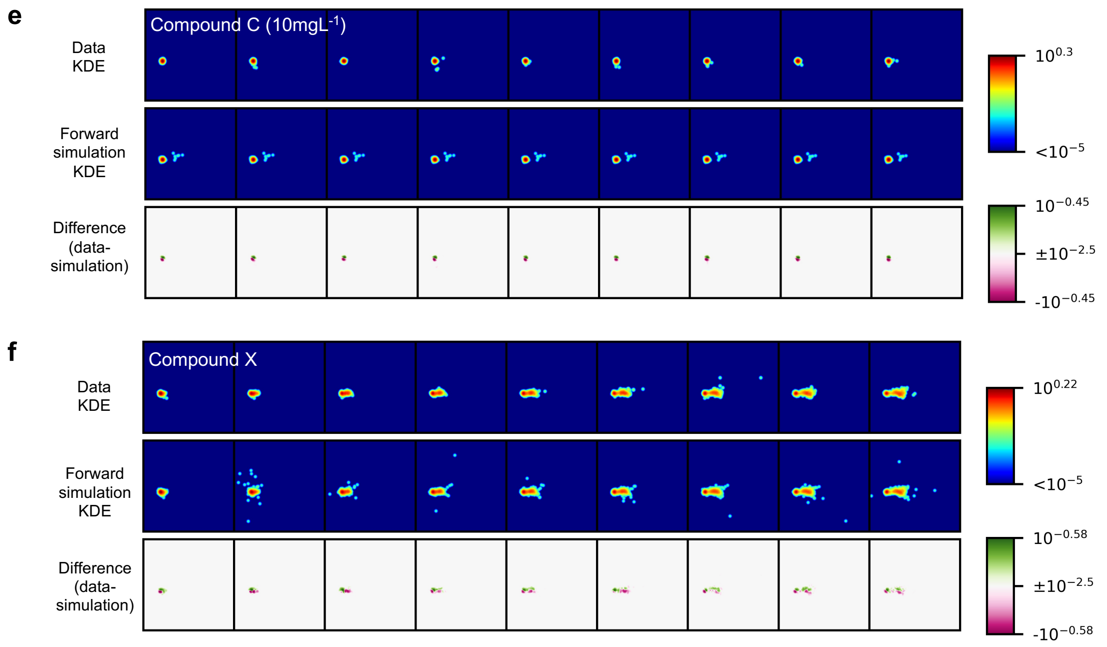

Supplementary Figure 3: **Validation of the landscapes and diffusivities learned by the PINN.** For each condition (**a-f**), three panels are shown: the first panel is the data kernel density estimate (KDE), the second is the KDE over simulations, and the third is the error (data KDE - simulation KDE). All are displayed on a logarithmic scale, and the error is truncated at $10^{-2.5}$, which is the probability density generated by a single particle, in order to highlight more systematic errors. For the forward simulations, particle starting positions were sampled from the initial probability distribution learned by the PINN, and then simulations were run by evaluating the potential and diffusivity on a 1000×1000 spatial grid, with 20 snapshots in time for the diffusivity. The figure shows good agreement across all conditions, validating the landscapes and diffusivities learned by the PINN.

Supplementary Figure 4: Comparison of simulation and data trajectories

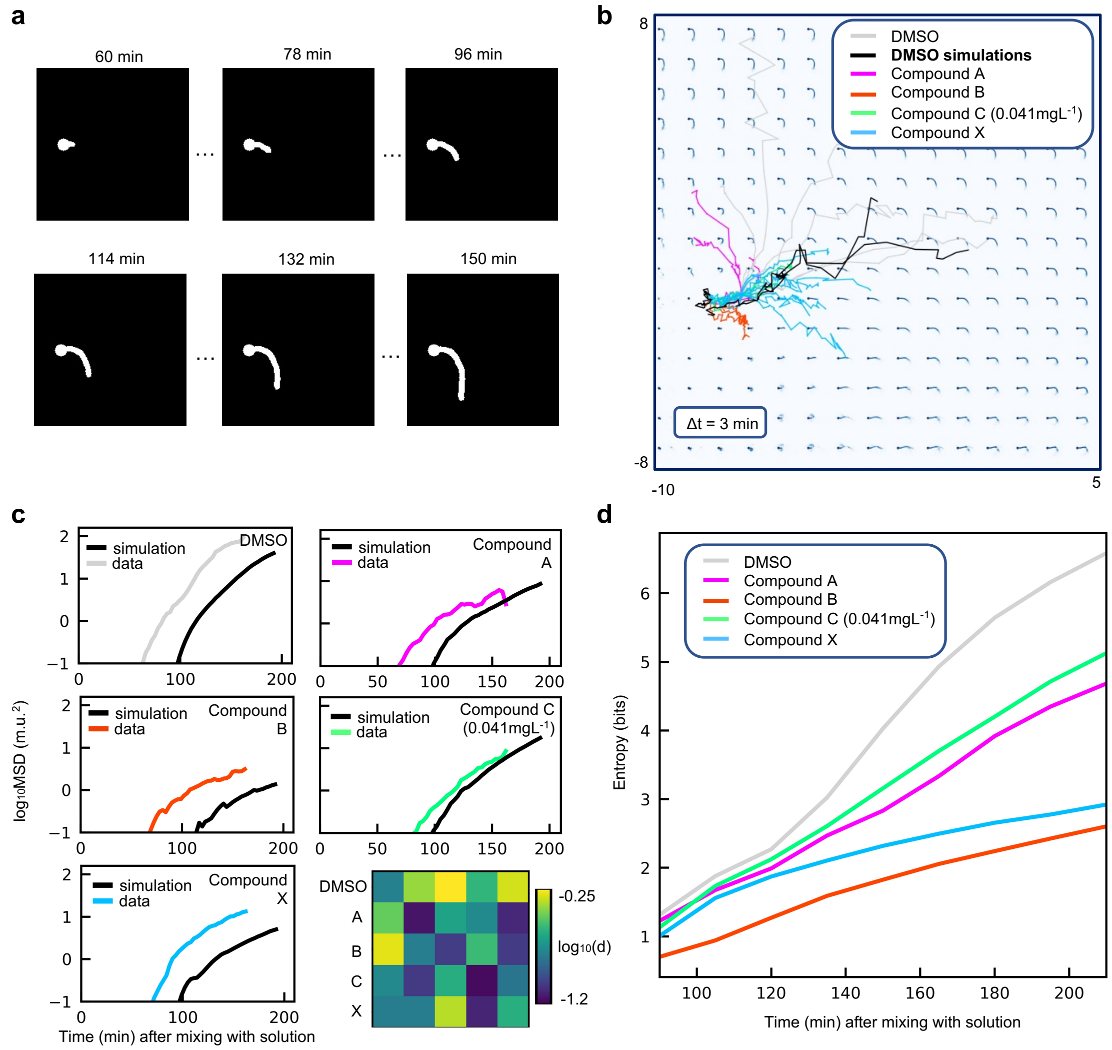

Supplementary Figure 4: **Comparison of simulation and data trajectories.** (**a**) An example sequence from the DMSO time-lapse videos. Images were taken every 3 min, from 60 min after mixing with the compounds. (**b**) Trajectories of sequential frames of the time-lapse videos (colored) and a sample of DMSO simulations (black). For the forward simulations, particle starting positions were sampled from the initial probability distribution learned by the PINN, and then simulations were run by evaluating the potential and diffusivity on a 1000×1000 spatial grid, with 20 snapshots in time for the diffusivity. (**c**) Mean squared displacement (MSD, in terms of morphospace units, m.u.) plots against time for the time-lapse videos (colored) and forward simulations (black). Time-lapse videos were taken under higher temperatures, which results in early germination. A confusion matrix of the mean absolute differences (d) of the plots is also shown. For each simulation-data pairing (videos down the rows, simulations across columns), the time series were shifted horizontally and the result for each pairing taken to be the minimum of the mean absolute differences across the shifts. Simulations match their corresponding data generally the best, except for the simulations of Compound X. (**d**) The entropy of PDFs from a KDE over single particle simulations of Eq. 1 reveals that entropy always increases with time. Entropy is calculated as $-\sum_{x_1}\sum_{x_2} p(\mathbf{x}) \log_2 p(\mathbf{x}) \Delta x_1 \Delta x_2$ with $\mathbf{x} = (x_1, x_2)$ and only summing over morphospace regions where the PDFs are above 10^{-3}. Source data are provided for (**c-d**).

Supplementary Figure 5: Data-driven development of the tip growth model

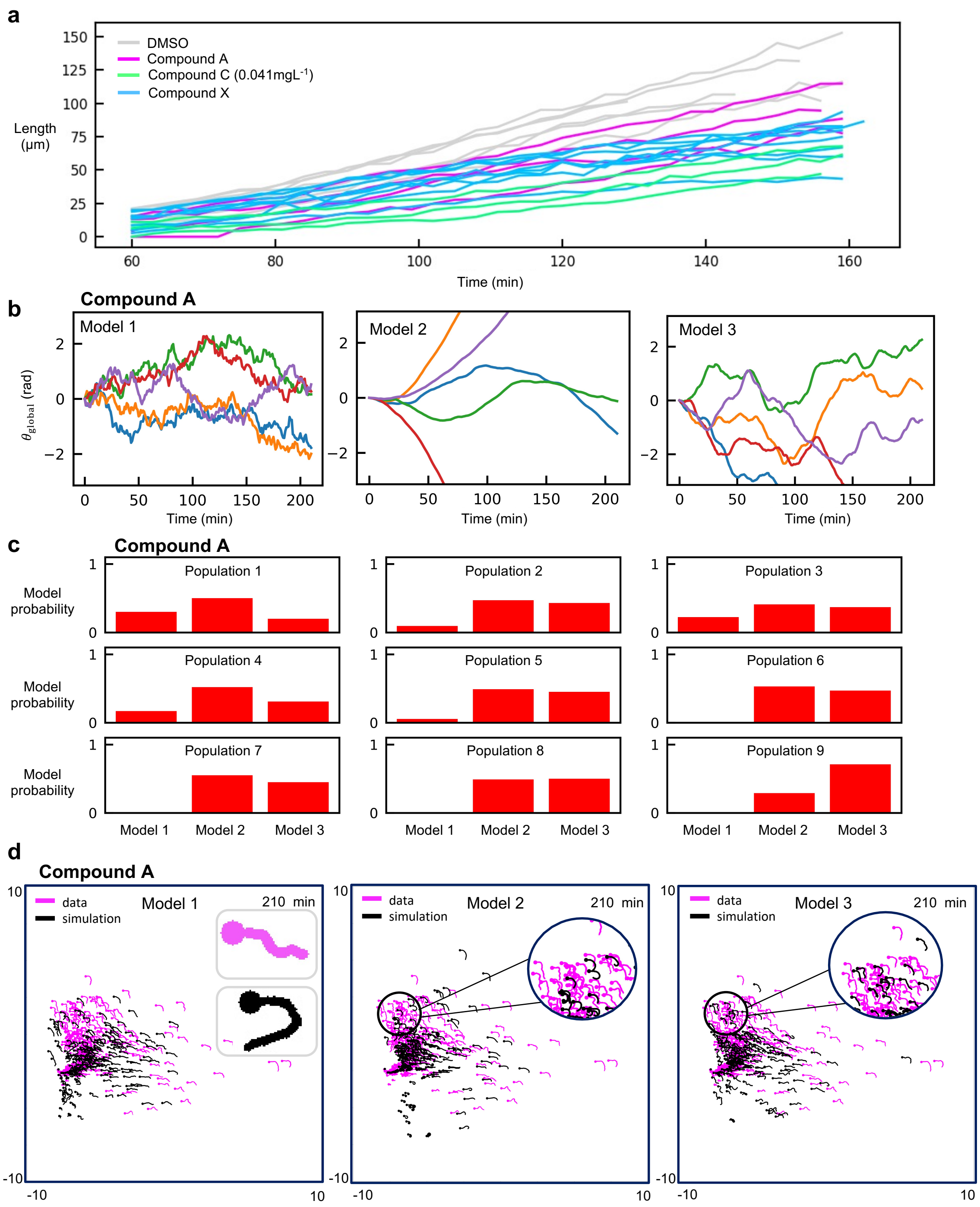

Supplementary Figure 5: **Data-driven development of the tip growth model.** Caption continued on the following page.

Supplementary Figure 5: **Data-driven development of the tip growth model.** (**a**) Time-lapse video data shows length increasing approximately linearly with time for all conditions. (**b**) Variation in global direction, θ_{global}, for the three tip bending models tested (using bending MAP values for Compound A, with a growth rate of 0.75 μm min^{-1}). Model 1 is a random walk in θ_{global}, Model 2 is a random walk in path curvature, κ, and Model 3 is a persistent random walk in κ, with relaxation to straight growth. (**c**) Model selection using ABC-SMC. For early populations where the acceptance threshold is high, the lower dimensional parameter spaces of models 1 & 2 lead to better fits. At lower acceptance thresholds, however, models 2 & 3 fit better, validating model conception in the tip frame, and ultimately the relaxation to straight growth in Model 3 is required to reproduce the data distribution. (**d**) Comparison of Compound A snapshot data (pink) and MAP simulations (black) for the three models at 210 min, with an enlarged example of a randomly selected simulation and data fungus shown in the inset of the Model 1 box. While all models introduce bending too early for some fungi (the region below the spore where there are simulations but no data), Model 3 can reproduce the feature distribution best. In particular, it is the only model that can reproduce fungi with multiple bends in alternating directions. Overall, this feature is less well separated in this 2D morphospace that prioritizes global features. Source data are provided for (**a**, **c**).

Supplementary Figure 6: Comparisons of maximum *a posteriori* probability (MAP) simulations of Model 3 with data

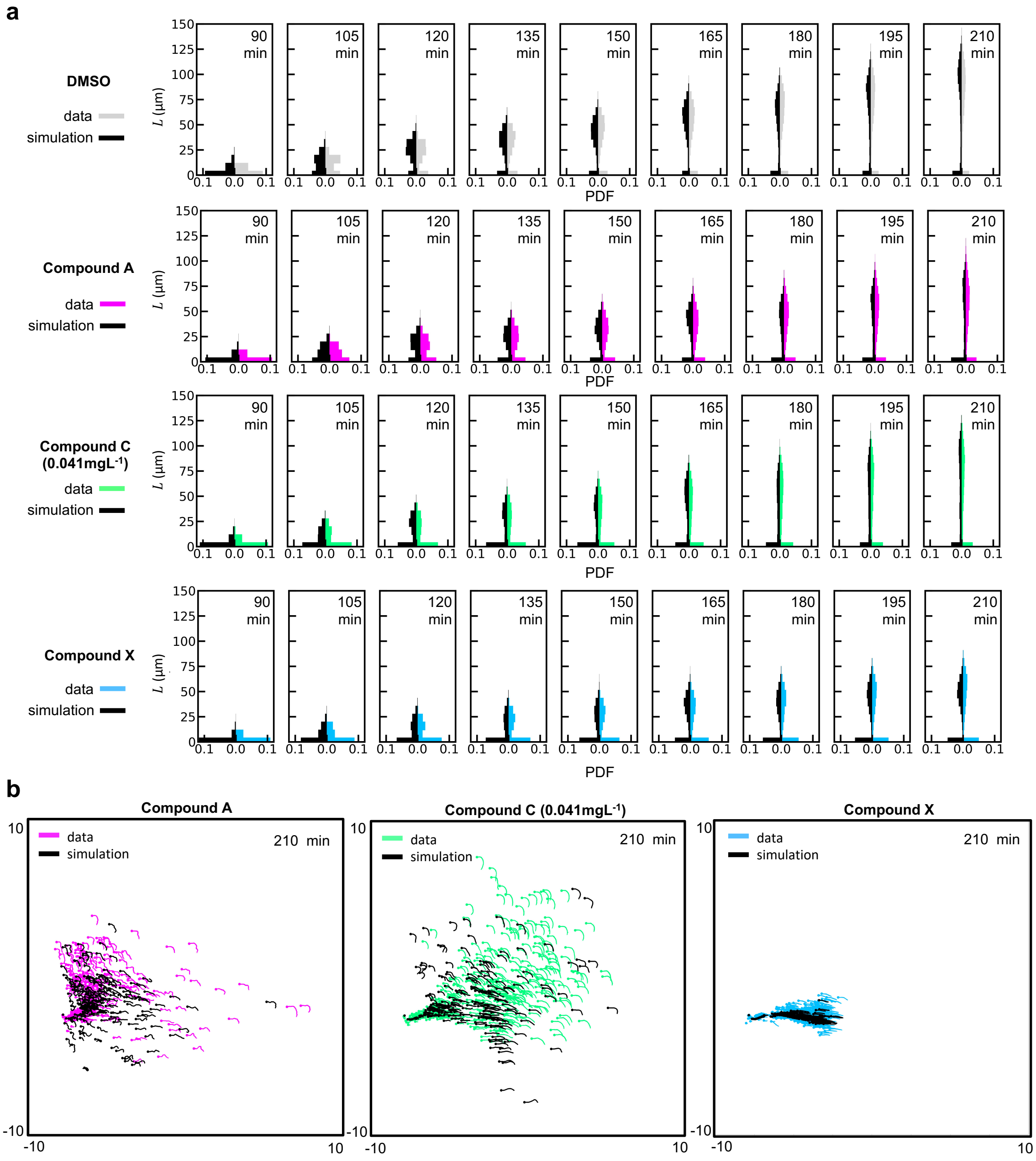

Supplementary Figure 6: **Comparisons of maximum *a posteriori* probability (MAP) simulations of Model 3 with data.** (**a**) Comparisons of the length distributions for the snapshot data (colored) and simulations (black) with MAP parameters for all conditions, showing good agreement. (**b**) Comparisons of snapshot data (colored) and simulations (black) with MAP parameters at 210 min, showing good agreement for tip bending. Source data are provided for (**a**).

Supplementary Figure 7: Chemical structure of Compound X

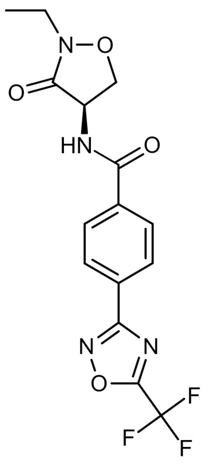

Supplementary Figure 7: **Chemical structure of Compound X.**

Supplementary Notes

Supplementary Note 1: *P. pachyrhizi* Imaging and Image Processing

P. pachyrhizi spore propagation

Urediniospores of the plant pathogenic basidiomycete *P. pachyrhizi* Syd. & P. Syd were prepared in a biosafety class 2 laboratory at Syngenta Stein, Switzerland, as follows: Glycine max (var. Toliman) plants were grown for 22-25 days in 8 cm pots in substrate with fertilizer. On the day before infection, all leaves except the second trifolium were cut, and plants were watered well. Approx. 30 mg of freshly harvested urediniospores of strain K8108 were suspended in 5 mL of 0.05 % (v/v) Tween 20 solution in sterile water, and the suspension was diluted to a final concentration of $5{\times}10^4$ spores per mL in 100 mL Tween solution. The plants were inoculated inside a sterile bench by spraying the bottom sides of the leaves evenly using an airbrush. Infected plants were then incubated at 22 °C, >90 % relative humidity, in the dark. 24 h post infection, the plants were placed back under light (20 K Lux, Metal Halid) at 22 °C day / 20 °C night conditions, 70 % relative humidity, and watered every 3 d. Once pustules (uredinia) were formed (usually 10 days post infection) the leaves were cut off and placed inside large Petri dishes containing a moisturized paper towel. The Petri dishes were closed with Parafilm and incubated for 3 to 4 d in the dark at room temperature. The spores were then detached from the upside-down held leaves inside a biosafety class 2 cabinet by gentle tapping, collected on sterile aluminum foil and used for microscopy assays on the same day.

P. pachyrhizi in vitro germination assay for snapshot and time-lapse imaging

Chemicals tested were either provided by Syngenta or purchased from Merck KGaA (Darmstadt, Germany) or other vendors as indicated. Stock solutions were prepared at 10 gL^{-1} in DMSO (Fisher Chemical, D/4121/PB15): Carbendazim (CAS-number 10605-21-7; Merck, 45368-250MG), PIK-75 (2-Methyl-5-nitrobenzenesulfonic acid [(6-bromoimidazo[1,2-a]pyridin-3-yl)methylene]methylhydrazide hydrochloride; CAS-number 372196-77-5; Angene AG00C6HA / AGN-PC-0RDGQ1; stock solution stored frozen at -80 °C in small aliquots), Compound X (Syngenta research compound related to trifluoromethyloxadiazoles [1]), Benzovindiflupyr (CAS-number 1072957-71-1; Syngenta). All compounds tested were diluted to two-fold the final concentration in water, either directly from the concentrated stock solutions or from serial 3-fold dilutions in DMSO. Assay plates in 96-well format were prepared before the spore suspensions by pipetting 50 µL of two-fold concentrated treatment solution into the respective wells of CellCarrier-96 Ultra plates (PerkinElmer, 6055302). Approx. 20 mg of freshly harvested *P. pachyrhizi* urediniospores were suspended in 2 mL of 0.015 % (v/v) Tween 20 in sterile water by shaking in a 5 mL vial (Axygen, SCT-5ML-S).

After determining the spore concentration by using a hemocytometer (Neubauer improved), the required volume of two-fold concentrated suspension with 20,000 spores per mL in 0.0015 % Tween 20 was prepared. The assay was started immediately by mixing 50 µL of spore suspension with treatment solution in the 96-well plates prepared in advance, resulting in a final one-fold concentration of the test compounds (Carbendazim: 1.1 mgL^{-1}; PIK-75: 3.3 mgL^{-1}; Compound X: 1.1 mgL^{-1}; Benzovindiflupyr: 0.041 and 10 mgL^{-1}) and 1000 spores per well, in a final DMSO concentration of 0.1 % in all wells. The plates were then incubated in a closed box at 20 °C in the dark. After 9 different incubation times between 90 and 210 min in 15 min intervals, 25 µL Calcofluor White staining solution (Merck, 18909-100ML-F) and 25 µL KOH 10 % (w/v) were added to each well to stain the fungal cell walls and simultaneously kill the germlings to enable snapshot imaging. The plates were sealed with adhesive aluminium PCR sealing foil (Thermo Scientific, AB-0626), mixed well by vortexing, and left at least for 20 min at room temperature to ensure consistent staining and to allow the spores to settle.

Imaging on the Opera QEHS High-Content Screening System (PerkinElmer) was performed with the following settings for excitation of Calcofluor White fluorescence (cell walls of germ tubes): 405 nm laser (350 µW), 10x air objective lens, 40 ms exposure time, camera bandpass filter 450/50 nm, pixel binning 2. For the excitation of autofluorescence of spore hulls (to differentiate them from germ tubes and appressoria) the following settings were used: 488 nm laser (15900 µW), 10x air objective lens, 1,600 ms exposure time, camera bandpass filter 565/40 nm, pixel binning 2. Twelve technical replicate wells were imaged per condition, and 47 images were taken per well at different positions.

Germination time-lapse imaging was performed on the JuLI Stage Real-Time Cell History Recorder (NanoEnTek Inc.). The germination assay was set up as described above, but after mixing spores with treatment solutions the assay plates were incubated directly in the imaging device, which itself was placed inside of a climate cabinet set to 20 °C. Imaging started with an autofocus run, followed by 321 runs with 3 min intervals to cover germination between +1 and +17 h after setup. No replicate wells were prepared to minimize interval times, but 5 different spots were imaged per well. The transmission light LED and the 10x objective were used with settings: 15 ms exposure time, LED power "3", brightness correction "10".

Image Processing

The size of the snapshot image sets necessitated fully-automated processing. We extracted fungus contours using adaptive binarization, where the threshold value varies based on the statistics of a surrounding window. We used 100×100 windows (the approximate size of lighting defects) out of full images of 503×685 pixels, and the threshold point was found from the cross correlation of this area with a Gaussian window, and shifted with biases of 30 and 0 out of the [0, 255] pixel range for the germ tube and spore images, respectively (found through trial and error). See the *adaptiveThreshold* function in OpenCV. This yielded two images per view: one with spore contours, and another with germ tube contours. Adding these together then gave an image with full fungi. Contours in this combined image with an area above a threshold found by trial and error were removed as obvious overlapping fungi, and the remainder were cropped by finding the minimum bounding rectangle (using OpenCV's *minAreaRect* function), and rotating to align with the pixel grid (using OpenCV's *warpAffine* function with bilinear interpolation) with padding so all were 200×200 regions of interest (ROIs), to fit the largest fungi in the set. Incomplete fungi were also removed by detecting if any of their outline points touched the image border.

We then used a supervised convolutional neural network to remove overlapping morphologies, given a set of hand-labelled examples. Single-fungus images were then translated and rotated so the initial growth directions coincided, and a flip was executed if the right-most point of the fungus was higher than the germination point. To do this alignment, we utilized the fact that there was one image of the spores, and another of the germ tubes for each view, and that these two contours overlapped slightly. The fungi were all translated so the points at which the germ tube hit the spore coincided. This point was found to be the center of mass of the overlap of the spore and germ tube contours. Since the germ tubes grow out perpendicular to the spore surface, alignment of the initial germination direction was then achieved by rotating the line joining the two points where the germ tube contour hit the spore to be vertical. These two points were found by tracing from the point on the germ tube contour furthest from the spore - germ tube intersection point in opposite directions until the spore was hit. We replaced all spores with identical circles, so as to prioritize modeling of the germ tube; the resulting morphospace point is then widened into a spore region through the kernel density estimation.

We removed overlapping fungi, which we note means larger morphologies are more likely to be removed than smaller ones (e.g. ungerminated spores), leading to a slight bias towards smaller morphologies. However, the DMSO data shows that there is very little germination after the first snapshot, which would lead to an artificially increasing percentage of spores if the bias was significant, which Supplemetary Fig. 1c does not show; instead, the inter-snapshot variability is the much more dominant factor. In other words, the variance in the latent variables that cause inter-snapshot variability dominates over the slight bias induced by removing overlapping fungi. In future, however, this slight bias can be resolved by using more sophisticated segmentation algorithms that can resolve overlapping morphologies [2], which would also increase the amount of data extracted per image.

For the time-lapse videos, we again binarized (using trial and error for a suitable threshold value, and without adaptive binarization, since there were not significant lighting defects), and found the fungus contours. We then found series of contours across frames whose center of mass were closest, and manually looked through these to find those that corresponded to tracking an individual fungus. We then used ImageJ to color over the spore with the background color (black background, white fungi), and aligned all germination points (found simply as the intersection point on the initial full-fungus contour image and the new contour image with the germ tube only). We then manually rotated and (where neccessary) flipped the images using Gimp, to ensure the initial growth directions coincided. Before being inputted into the autoencoder, snapshot and time-lapse video pixels were assigned to a value in the set $\{0, 1\}$.

Supplementary Note 2: Autoencoder and PINN Neural Networks

Autoencoder architecture and training

For the autoencoder's encoder, we used four convolutional layers with 16, 32, 64 and 16 feature maps, all with 3×3 kernels, ReLU activations, batch normalization, and alternating stride sizes of 1 and 2. The decoder's structure mirrored the encoder's, but with transposed convolutions. We used a sigmoid output activation and binary cross entropy loss, over mini-batches of 50 images, and trained for 4 epochs using the Adam optimizer [3] with a learning rate of 10^{-4}, which took 2 hours with a Quadro RTX 6000 GPU card. Training was stopped at the point at which the trajectories of the single-fungus videos were least complex.

Principal Component Analysis (PCA) is a commonly-used linear dimensionality technique that finds a set of orthogonal features based on the data variance in different directions. While this method can find very interpretable features, non-linear methods can be more expressive, capturing curved data manifolds to describe the data in fewer dimensions. This non-linearity is particularly useful for our work, as it allows us to find a 2D morphospace, which then enables intuitive visualization of the Fokker-Planck model landscapes.

t-distributed stochastic neighbor embedding (t-SNE, [4]) is another commonly-used non-linear dimensionality reduction algorithm that could be used for finding the morphospace, and for characterizing morphodynamics with the Fokker-Planck model. However, this algorithm is non-parametric, meaning that when new data is added, it must be re-run in full, and so is not well-suited for the implementation of approximate Bayesian inference on mechanistic model parameters.

If the autoencoder is too small (i.e. shallow or narrow), the embedding function may be too simple to capture curved data manifolds. For example, autoencoders with single hidden layer and linear activation functions can only learn linear features. However, if the network is too large, it can reconstruct the data without needing to closely describe the underlying manifold. This can be thought of as a form of overfitting. For higher-dimensional latent spaces, this can be a more significant problem, and so autoencoder variants that regularize the latent space are typically used, for example the Variational Autoencoder (VAE) [5], which imposes a Gaussian prior distribution over the latent space. These ensure the latent space is smooth, with meaningful features throughout. We found that the autoencoder architecture used here was suitable for capturing the data manifold, and avoided variants like the VAE because they warped fungus trajectories that were more natural with a standard autoencoder.

Learning landscapes from snapshot data

For the PINN, the loss function to be minimized comprizes four terms, with the first three calculated over random mini-batches of N data points, and the final one over the full spatial grid of M data points. The first is the mean squared difference between the learned PDF, $\hat{p}(\mathbf{x}^j, t^j)$, and data, $p(\mathbf{x}^j, t^j)$,

$$L_{\mathrm{PDF}} = \frac{1}{N}\sum_{j=1}^{N}\left[\hat{p}(\mathbf{x}^j, t^j) - p(\mathbf{x}^j, t^j)\right]^2, \tag{S1}$$

with $\{\mathbf{x}^j, t^j\}$ in the nine snapshots. The second is the mean squared PDF at the boundary,

$$L_{\mathrm{BC}} = \frac{1}{N}\sum_{j=1}^{N}\left[\hat{p}(\mathbf{x}^j, t^j)\right]^2 \tag{S2}$$

with $\{\mathbf{x}^j, t^j\}$ selected from 10^6 uniformly distributed boundary points, and the third term is the mean squared PDE residual ($\mathcal{N}$, given in Eq. 3),

$$L_{\mathrm{PDE}} = \frac{1}{N}\sum_{j=1}^{N}\left[\mathcal{N}(\hat{p}(\mathbf{x}^j, t^j), \hat{D}(\mathbf{x}^j, t^j), \hat{U}(\mathbf{x}^j))\right]^2, \tag{S3}$$

with $\{\mathbf{x}^j, t^j\}$ selected from 10^6 points uniformly distributed over the whole domain. The final term ensures the PDF integrates to one:

$$L_{\mathrm{norm}} = \left[\sum_{j=1}^{M}\Delta x_1 \Delta x_2 \hat{p}(\mathbf{x}^j, t) - 1\right]^2, \tag{S4}$$

with $\mathbf{x}^j$ covering the full spatial grid and t randomly selected.

For the total loss (Eq. 4), we used hyperparameters of 1, 1, 500, 0.01 for a, b, c and d. Previous work using PINNs to solve the Fokker-Planck equation [6, 7] used a weighting for L_{PDE} of 100, and the same values for the other hyperparameters as we used. Since our data was of snapshots of different spore batches, we increased the weighting to 500, to prioritize more the PDE fitting over the data. While initially selected by trial and error through inspecting single particle forward simulations, this choice was later vindicated quantitatively. We trained the DMSO PINN with c values of 50, 500 and 5000 for 10 hours each. Since changing the hyperparameters modifies the loss calculation, L_{total} cannot be used to test the quality of the inferred parameters. Instead, we compared the resulting fits of single particle simulations of Eq. 1 with the data through the mean absolute probability error on the grid (MAE). Particle starting positions were sampled from the initial PDF learned by the PINN, and then simulations were run by evaluating the potential and diffusivity on a 1000×1000 spatial grid, with 20 snapshots in time for the diffusivity, with a time step of 0.01 min. The MAE values for c values of 50, 500 and 5000 were 9.8×10^{-6}, 9.0×10^{-6} and 9.9×10^{-6} respectively, showing 500 was a suitable choice for c, as it produced the model with the best fit to the data. Reducing the relative weighting of the data penalty means there is no longer a guarantee of the learned PDF integrating to one, and so a normalization penalty is required. The weighting for L_{norm} of 0.01 was found to normalize the data adequately, without excessively prioritising this constraint, in agreement with the findings of [6, 7].

Since *P. pachyrhizi* were fixated upon staining, the snapshots were of distinct populations. There are therefore latent variables that change the dynamics for each population. Ideally, the learned solution would be the average dynamics of infinite repeats. Since neural networks learn smooth low-frequency solutions first, we used early stopping to capture average dynamics, rather than letting the dynamics fit exactly to each snapshot, primarily watching for when the PINN began to fit individual snapshots. This point approximately corresponded to 30, 30, 30, 20, 25 and 25 hours for DMSO and Compounds A, B, C (0.041 mgL^{-1}), C (10 mgL^{-1}) and X, respectively. For small numbers of snapshots, it is possible that spurious patterns emerge not only on the single-snapshot level, but also across many snapshots; for example if two subsequent snapshots have similar low-probability latent variables, this will be captured by the PINN. However, such patterns become increasingly less problematic as the number of snapshots increases. An alternative solution is to constrain the solution physically, as we did with the tip growth model.

The three neural networks had 5 fully connected layers, each with 50 neurons, with residual skip connections, and swish activations between layers. A softplus output activation was used for the PDF, and sigmoid was used for the potential and diffusivity, with the potential multiplied by 3 to give a [0, 3] output range. This sigmoid constraint prevents unphysical solutions that can arise with unbounded force and diffusivity. Output variables that share inputs (e.g. the PDF and diffusivity) can be outputted from a single neural network if they are likely to have similar features, for increased computational efficiency. We used the Adam optimizer [3] with a learning rate of 5×10^{-4}, and batch sizes, N, of 8,000. To speed up training, the DMSO landscape was first trained for 10 hours, and PINNs for the other conditions were initialized with these weights (known as transfer learning).

The landscapes for DMSO and Compound A formed deep bowls at the outer morphospace regions. For visualization, we therefore scaled landscape regions outside the PDF boundary of 10^{-3} towards the closest point on this boundary and applied a Gaussian smoothing to this outer region. This way, the full landscape can be easily compared with the morphospace, but with decreased gradients in regions where there is not significant data density. This is a purely cosmetic choice, and the original landscapes can also be viewed unedited, since the translucency does enable viewing of valley regions.

There are two sources of stochasticity in the fungus trajectories over the landscapes, which we call morphodynamic diffusion and embedding noise, both of which are consumed into the diffusion term of the Fokker-Planck model. Morphodynamic diffusion is the fundamental unpredictability of morphodynamics over time, arising from un-modeled internal and external factors that vary across fungi. This corresponds to diffusion over the approximately 2D morphological manifold within the high-dimensional pixel space. Embedding noise arises from fungi being randomly perturbed away from the 2D morphology manifold in the high-dimensional pixel space. Such perturbations arise from factors including image resolution, segmentation and alignment (which for example add extra degrees of freedom to the images, and each degree of freedom expand the data manifold dimensionality), and the complexity of the autoencoder's embedding function means these perturbations are not always just mapped to the closest point on the manifold. Since the same

image pre-processing algorithms were used on all conditions, differences in the Fokker-Planck diffusion over the same region of morphospace will be morphodynamic in nature, rather than arising from the embedding noise.

Supplementary Note 3: Three Models of Tip Growth

The minimal model for fungus growth was composed of two equations: one for lengthening and another for tip bending, and we ran model selection on three candidate models for the bending part.

For fungus lengthening, we used the 3-parameter lognormal distribution to model both germination time, t_g, and growth rate, α. The probability density function of the 3-parameter lognormal distribution is given by

$$f(x; s, \sigma^2, \text{loc}) = \frac{1}{\sigma\sqrt{2\pi}(x - \text{loc})} \exp \frac{\log^2\left(\frac{x-\text{loc}}{s}\right)}{2\sigma^2} \tag{S5}$$

where σ is a shape parameter, s is a scale parameter (also the median), and loc is a location parameter (the lower bound). The 2-parameter distribution has loc set to zero.

We modeled germination time, t_g, as distributed according to $t_\text{g} \sim \textit{lognormal}(s_{t_\text{g}}, \sigma_{t_\text{g}}, \text{loc}_{t_\text{g}})$, and growth rate, α, as distributed according to $\alpha = \text{loc}_\alpha - x$, with $x \sim \textit{lognormal}(s_\alpha, \sigma_\alpha, 0)$ and resampling for negative α.

For inferring both the lengthening and bending parameters, we used ABC-SMC [8]. This is a computationally efficient implementation of ABC, identifying intermediate distributions over a series of populations, and gradually decreasing the acceptance threshold. All histograms were compared using the summed absolute distance, and we trained the autoencoder for an extra two epochs with simulations generated randomly from the prior distribution to get coverage of any novel features.

For inferring lengthening parameters (s_{t_g}, σ_{t_g}, loc_{t_g}, s_α, σ_α, loc_α), we ran ABC-SMC with a population size of 100, each with 5000 simulations, for 4 steps. All prior distributions were uniform distributions over the following ranges: s_{t_g}: [0.01, 150], σ_{t_g}: [0.01, 5], loc_{t_g}: [20, 100], s_α: [0.01, 2], σ_α: [0.01, 1], loc_α: [0.3, 1.5].

We compared three models for tip bending, and used ABC-SMC for both model selection and parameter inference. As described in the main text, the comparison was done using both the fungus length data and 210 min morphospace embeddings, and Model 3 was found to reproduce the data best. For all of the following, σ is a noise parameter that was fitted, and dW is the Wiener process. Model 1 was a random walk in the global direction, θ_global, a simple model commonly used in the literature:

$$d\theta_\text{global} = \sigma dW. \tag{S6}$$

Model 2 was a random walk in the curvature of the growth path, κ, in order to connect to cell tip mechanics:

$$d\kappa = \sigma dW. \tag{S7}$$

Model 3 was a persistent random walk in the curvature, with an additional parameter for relaxation to straight growth, τ^{-1}, motivated by work analysing fission yeast tip growth mechanics [9]:

$$d\kappa = -\tau^{-1}\kappa dt + \sigma dW. \tag{S8}$$

Models 2 and 3 can be loosely connected to a diffusing growth zone at the tip as has been described in fission yeast [9], by introducing an angular growth zone position, θ_tip, and a mapping, $\kappa = f(\theta_\text{tip})$, where f is unknown, but likely monotonically increasing, and passing through the origin (i.e. a central growth zone corresponds to straight growth).

For fitting the bending parameters using ABC-SMC, images were created by taking the MAP lengthening parameters, running the model, and then converting to Cartesian coordinates using the relation $\frac{d\theta_\text{global}}{dt} = \frac{dL}{dt}\frac{d\theta_\text{global}}{dL} = \alpha\kappa$, such that:

$$x_{N+1} = x_N + \alpha\Delta t \cos\left(\sum_{n=0}^{N} \alpha\kappa\Delta t\right) \tag{S9}$$

$$y_{N+1} = y_N + \alpha\Delta t \sin\left(\sum_{n=0}^{N} \alpha\kappa\Delta t\right). \tag{S10}$$

Coordinates were then converted to images using the *polylines* function in OpenCV.

For selecting the optimal bending model, we ran ABC-SMC with a population size of 40, each with 1000 simulations, for 9 steps. All prior distributions were uniform distributions over the following ranges: Model 1, σ: [0, 0.2]; Model 2, σ: [0, 0.01]; Model 3, σ: [0, 0.05], τ^{-1}: [0, 0.2].

For subsequently inferring the bending parameters of Model 3, we used a population size of 40, each with 1000 simulations, and ran for 4 steps using the same prior distributions as for model selection.

Supplementary Note 4: Possible Modes of Action

The compounds were identified at pre-screening by eye to show a range of phenotypes.

Compound A (methyl benzimidazol-2-ylcarbamate) is a widely-used fungicide that inhibits the assembly of tubulin subunits into functional microtubules, which are an essential part of the cytoskeleton [10]. Microtubules participate in maintaining the shape of cells, the distribution of organelles, the transport of materials within the cell, and in the separation of chromosomes during mitosis. The bending of germ tubes that we observe may be in part induced by disturbed vesicle transport towards the growing hyphal tip, although there is likely a range of causes.

Compound B (PIK-75 hydrochloride) is a phosphoinositide 3-kinase (PI3K) inhibitor. PI3Ks are components of certain signaling pathways, which control growth, metabolism and other functions. PIK-75 comes from the pharmaceutical industry and is technically not a fungicide. The fungal target is unknown, but if it inhibits fungal PI3K, then the observed phenotype could be due to the disturbance of cellular signaling required for normal tip growth.

Compound C (benzovindiflupyr) has complex II of the respiratory chain as the target, and consequently an inhibition can lead to a depletion of energy. Therefore, depending on the concentration, the cell will stop growth because it lacks the ability to produce the required metabolites.

Compound X (a Syngenta research compound related to trifluoromethyloxadiazoles [1], see Fig. 7 for the chemical structure) is an inhibitor of a histone deacetylase (HDAC). Histones are proteins that interact with nuclear DNA, and HDACs are enzymes that remove acetyl groups from histones. As a result, the DNA is packed more tightly, which has an influence on gene expression. HDAC-inhibitors result in de-regulation of this control mechanism for gene expression, however it is unknown why *P. phakopsora* germlings react to this compound in the observed manner.

Supplementary Methods

Python was used for all computing, with the OpenCV library [11] used for processing the snapshot images, and for data-driven development of the tip growth model. We manually built the PINN architecture using Tensorflow 2 [12], however there is now a full PINN Python package, DeepXDE [13]. A Quadro RTX 6000 GPU card was used to speed up neural network training and inference. Finally, we used the pyABC library [14] for parameter fitting of the tip growth model with ABC-SMC, the computationally efficient implementation of approximate Bayesian computation.

Supplementary References

[1] C. Winter et al. "Trifluoromethyloxadiazoles: inhibitors of histone deacetylases for control of Asian soybean rust". In: *Pest Management Science* 76.10 (2020), pp. 3357–3368.

[2] Y. Song et al. "Accurate cervical cell segmentation from overlapping clumps in pap smear images". In: *IEEE transactions on medical imaging* 36.1 (2016), pp. 288–300.

[3] D. P. Kingma and J. Ba. "Adam: A method for stochastic optimization". In: *arXiv preprint arXiv:1412.6980* (2014).

[4] L. Van der Maaten and G. Hinton. "Visualizing data using t-SNE." In: *Journal of machine learning research* 9.11 (2008).

[5] D. P. Kingma and M. Welling. "Auto-encoding variational bayes". In: *arXiv preprint arXiv:1312.6114* (2013).

[6] Y. Xu et al. “Solving Fokker-Planck equation using deep learning”. In: *Chaos: An Interdisciplinary Journal of Nonlinear Science* 30.1 (2020), p. 013133.

[7] X. Chen et al. “Solving inverse stochastic problems from discrete particle observations using the fokker-planck equation and physics-informed neural networks”. In: *arXiv preprint arXiv:2008.10653* (2020).

[8] T. Toni et al. “Approximate Bayesian computation scheme for parameter inference and model selection in dynamical systems”. In: *Journal of the Royal Society Interface* 6.31 (2009), pp. 187–202.

[9] T. Drake and D. Vavylonis. “Model of fission yeast cell shape driven by membrane-bound growth factors and the cytoskeleton”. In: *PLoS Comput Biol* 9.10 (2013), e1003287.

[10] N. Takeshita and R. Fischer. “On the role of microtubules, cell end markers, and septal microtubule organizing centres on site selection for polar growth in Aspergillus nidulans”. In: *Fungal biology* 115.6 (2011), pp. 506–517.

[11] G. Bradski and A. Kaehler. *Learning OpenCV: Computer vision with the OpenCV library.* ” O’Reilly Media, Inc.”, 2008.

[12] M. Abadi et al. “Tensorflow: A system for large-scale machine learning”. In: *12th {USENIX} symposium on operating systems design and implementation ({OSDI} 16)*. 2016, pp. 265–283.

[13] L. Lu et al. “DeepXDE: A deep learning library for solving differential equations”. In: *SIAM Review* 63.1 (2021), pp. 208–228.

[14] E. Klinger, D. Rickert, and J. Hasenauer. “pyABC: distributed, likelihood-free inference”. In: *Bioinformatics* 34.20 (2018), pp. 3591–3593.
