# Supplementary material for: Physics-informed deep learning characterizes morphodynamics of Asian soybean rust disease
Source: Nat Commun. 2021 Nov 5;12:6424. doi: 10.1038/s41467-021-26577-1 (PMC8571353; doi:10.1038/s41467-021-26577-1)
Supplement: Supplementary file 3 — Description of Additional Supplementary Files [file 41467_2021_26577_MOESM3_ESM.pdf]

Description of Additional Supplementary Files

File Name: Supplementary Movie 1
Description: Phakopsora pachyrhizi germinating in DMSO imaged with the JuLI Stage Real-Time Cell History Recorder (NanoEnTek Inc.) at 20°C, frames every 3 min, 60 min after mixing with the compound.

File Name: Supplementary Movie 2
Description: Phakopsora pachyrhizi germinating in compound A (1.1 mgL^{-1}), imaged with the JuLI Stage Real-Time Cell History Recorder (NanoEnTek Inc.) at 20°C, frames every 3 min, 60 min after mixing with the compound.

File Name: Supplementary Movie 3
Description: Phakopsora pachyrhizi germinating in compound B (3.3 mgL^{-1}), imaged with the JuLI Stage Real-Time Cell History Recorder (NanoEnTek Inc.) at 20°C, frames every 3 min, 60 min after mixing with the compound.

File Name: Supplementary Movie 4
Description: Phakopsora pachyrhizi germinating in compound C (0.041 mgL^{-1}), imaged with the JuLI Stage Real-Time Cell History Recorder (NanoEnTek Inc.) at 20°C, frames every 3 min, 60 min after mixing with the compound.

File Name: Supplementary Movie 5
Description: Phakopsora pachyrhizi germinating in compound X (1.1 mgL^{-1}), imaged with the JuLI Stage Real-Time Cell History Recorder (NanoEnTek Inc.) at 20°C, frames every 3 min, 60 min after mixing with the compound.
