# Supplementary material for: Physics-informed deep learning characterizes morphodynamics of Asian soybean rust disease
Source: Nat Commun. 2021 Nov 5;12:6424. doi: 10.1038/s41467-021-26577-1 (PMC8571353; doi:10.1038/s41467-021-26577-1)
Supplement: Supplementary file 9 — Reporting Summary [file 41467_2021_26577_MOESM9_ESM.pdf]

nature research

Corresponding author(s): Robert G. Endres

Last updated by author(s): Sept 29, 2021

Reporting Summary

Nature Research wishes to improve the reproducibility of the work that we publish. This form provides structure for consistency and transparency in reporting. For further information on Nature Research policies, see our Editorial Policies and the Editorial Policy Checklist.

Statistics

For all statistical analyses, confirm that the following items are present in the figure legend, table legend, main text, or Methods section.

n/a	Confirmed	
☐	☒	The exact sample size (*n*) for each experimental group/condition, given as a discrete number and unit of measurement
☐	☒	A statement on whether measurements were taken from distinct samples or whether the same sample was measured repeatedly
☒	☐	The statistical test(s) used AND whether they are one- or two-sided<br>*Only common tests should be described solely by name; describe more complex techniques in the Methods section.*
☒	☐	A description of all covariates tested
☒	☐	A description of any assumptions or corrections, such as tests of normality and adjustment for multiple comparisons
☒	☐	A full description of the statistical parameters including central tendency (e.g. means) or other basic estimates (e.g. regression coefficient) AND variation (e.g. standard deviation) or associated estimates of uncertainty (e.g. confidence intervals)
☒	☐	For null hypothesis testing, the test statistic (e.g. F, t, r) with confidence intervals, effect sizes, degrees of freedom and P value noted<br>*Give P values as exact values whenever suitable.*
☐	☒	For Bayesian analysis, information on the choice of priors and Markov chain Monte Carlo settings
☒	☐	For hierarchical and complex designs, identification of the appropriate level for tests and full reporting of outcomes
☒	☐	Estimates of effect sizes (e.g. Cohen's *d*, Pearson's *r*), indicating how they were calculated

Our web collection on statistics for biologists contains articles on many of the points above.

Software and code

Policy information about availability of computer code

Data collection	Data was captured on the Opera QEHS running Opera Software 2.0 (EvoShell, Opera CHKN/QEHS Red Ver. 2.0.0.12017 Rev.: 89046, PerkinElmer Inc.) and on the JuLI Stage Real Time Cell History Recorder running JuLI Stage V. 2.0.1 and JuLI EDIT V. 1.0.0.0 (NanoEnTek Inc.).
Data analysis	Custom code was used (https://github.com/hcbiophys/morphodynamics).<br>Versions used:<br>ImageJ (Fiji) 2.1.0/1.53c<br>Gimp 2.10<br>python 3.7.3<br>torch 1.6.0<br>torchvision 0.2.1<br>tensorflow 2.3.1<br>numpy 1.20.2<br>opencv-python 4.4.0.46<br>pyabc 0.10.3<br>scipy 1.6.2<br>matplotlib 3.4.1<br>mayavi 4.7.2

For manuscripts utilizing custom algorithms or software that are central to the research but not yet described in published literature, software must be made available to editors and reviewers. We strongly encourage code deposition in a community repository (e.g. GitHub). See the Nature Research guidelines for submitting code & software for further information.

Data

Policy information about availability of data

All manuscripts must include a data availability statement. This statement should provide the following information, where applicable:

- Accession codes, unique identifiers, or web links for publicly available datasets
- A list of figures that have associated raw data
- A description of any restrictions on data availability

The image data that support the findings of this study have been deposited at http://cellimagelibrary.org/groups/54615 and figure source data are provided in an Excel document.

Field-specific reporting

Please select the one below that is the best fit for your research. If you are not sure, read the appropriate sections before making your selection.

☒ Life sciences ☐ Behavioural & social sciences ☐ Ecological, evolutionary & environmental sciences

For a reference copy of the document with all sections, see nature.com/documents/nr-reporting-summary-flat.pdf

Life sciences study design

All studies must disclose on these points even when the disclosure is negative.

Sample size	Approximately 600,000 single-fungus images were analyzed across snapshot times and compounds. This is the total number of single-fungus images that could be cropped from the original images of multiple fungi (Fig. 1a). These are qualitatively similar to the MNIST dataset of handwritten digits, with contains 60,000 images, and is commonly used for benchmarking dimensionality-reduction techniques in the machine learning literature. 600,000 was therefore a large enough number to estimate the probability distribution over shapes of growing P. pachyrhizi in 2D, as confirmed in Fig. 2.
Data exclusions	An extra compound was excluded as it induced features that could not be well-captured in a 2D shape space, which is mentioned in Discussion. Also discussed is future work accounting for this.
Replication	For Phakopsora pachyrhizi germination snap shot imaging, two replicate plates were prepared per incubation time, with each plate containing twelve replicate wells per treatment. All attempts at replication were successful, i.e. observed to give qualitatively similar phenotype distributions (compared by plotting different wells as done in Fig. 2c), and were therefore used in the downstream analysis. Replication was not performed with the time-lapse movies, because these were used to observe broad features of growth across conditions (e.g. linear growth, variable germination time), so in a way each condition served as a 'replicate' for these broad features, before fitting the model with the snapshot data. The time-lapse imaging is more labour-intensive and so running many replicates was not possible.
Randomization	The snapshot data is from germlings inactivated by chemical treatment after a defined incubation period with the phenotype-inducing compounds. The pipetting of spore suspensions, treatment and staining solutions was done manually, and the difference between the first and last wells of a plate was less than one minute at each step. As the samples were fixed before imaging, effects like the time required for imaging the whole plate and the temperature inside the microscope etc. were assumed to have no effect on the phenotypes of germlings. We are currently not equipped with a pipetting robot that would provide a randomized plate layout for chemical treatments, therefore manual pipetting of a pre-defined layout was the only option.
Blinding	Since the objects in our case are microorganisms, there was no placebo effect to be taken into account. As both the imaging (image positions per well pre-defined before the assay) and the image analysis were done in a non-subjective manner, blinding does not apply.

Reporting for specific materials, systems and methods

We require information from authors about some types of materials, experimental systems and methods used in many studies. Here, indicate whether each material, system or method listed is relevant to your study. If you are not sure if a list item applies to your research, read the appropriate section before selecting a response.

Materials & experimental systems

n/a	Involved in the study	
☒	☐	Antibodies
☐	☒	Eukaryotic cell lines
☒	☐	Palaeontology and archaeology
☒	☐	Animals and other organisms
☒	☐	Human research participants
☒	☐	Clinical data
☒	☐	Dual use research of concern

Methods

n/a	Involved in the study	
☒	☐	ChIP-seq
☒	☐	Flow cytometry
☒	☐	MRI-based neuroimaging

Eukaryotic cell lines

Policy information about cell lines

Cell line source(s)	The sequenced reference P. pachyrhizi strain K8108 is a single urediniosoral isolate from P. pachyrhizi-infected soybean leaves collected in 2015 in Uruguay (Colonia region). Genome sequence of this isolate is available at https://mycocosm.jgi.doe.gov/PhapaK8108/PhapaK8108.home.html
Authentication	The fungal cell line K8108 was generated by single spore isolation, without external authentication.
Mycoplasma contamination	P. pachyrhizi is a biotrophic fungal pathogen, cells were not tested for Mycoplasma contamination.
Commonly misidentified lines (See ICLAC register)	No misidentified lines were used in this study.
